# Solvent-Responsive Glass Transition Behavior of Polyelectrolyte Complexes

**DOI:** 10.1021/acs.macromol.4c02417

**Published:** 2024-12-23

**Authors:** Hongwei Li, Dmitry Tolmachev, Piotr Batys, Maria Sammalkorpi, Jodie L. Lutkenhaus

**Affiliations:** † Artie McFerrin Department of Chemical Engineering, Texas A&M University, College Station, Texas 77843, United States; ‡ Department of Chemistry and Materials Science, Aalto University, P.O. Box 16100, 00076 Aalto, Finland; § Academy of Finland Center of Excellence in Life-Inspired Hybrid Materials (LIBER), Aalto University, P.O. Box 16100, 00076 Aalto, Finland; ∥ Jerzy Haber Institute of Catalysis and Surface Chemistry, Polish Academy of Sciences, Niezapominajek 8, 30-239 Krakow, Poland; ⊥ Department of Materials Science and Engineering, Texas A&M University, College Station, Texas 77840, United States

## Abstract

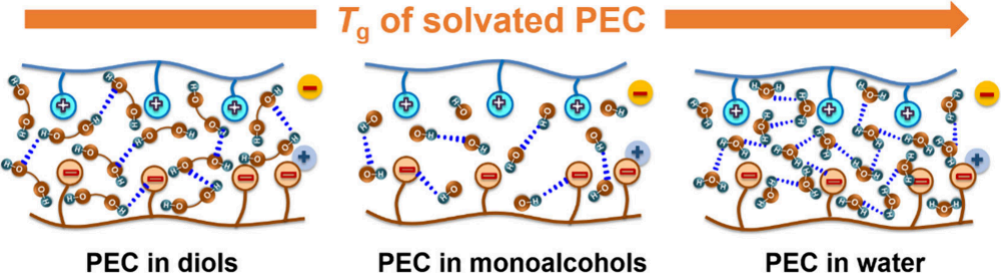

Polyelectrolyte complexes (PECs) have attracted considerable attention owing to their unique physicochemical properties and potential applications as smart materials. Herein, the glass transitions of PECs solvated with varying alcohols are investigated in poly(diallyldimethylammonium)/poly(acrylic acid) (PDADMA/PAA) complexes by using modulated differential scanning calorimetry (MDSC). Solvents with one or two hydroxyl groups are selected to examine the effect of PAA-solvent interactions on the glass transition temperature (*T*_g_). Except for glycerol, all alcohol solvents yield PECs with detectable *T*_g_’s and plasticization behavior. Furthermore, a linear relationship for 1/*T*_g_ and the natural logarithm of the number of hydroxyl groups to intrinsic ion pair ratio [ln(*n*_hydroxyl_/*n*_intrinsic-ion-pair_)] is found. This result is significant because prior work demonstrated the relationship only for water and no other solvents. All-atom molecular dynamics (MD) simulations analyze the ability of the solvent to form hydrogen bonds via the solvent’s OH groups to the PAA, revealing that the solvent molecule size and available hydroxyl groups govern the change in the glass transition. Overall, the clear dependence of a PEC’s glass transition on the solvent’s chemical structure provides a simple guideline for predicting their relationship.

## Introduction

Polyelectrolyte complexes (PECs) are a class of materials arising from the electrostatic interactions between oppositely charged polyelectrolytes (PEs), formed spontaneously in solution with counterion release and ion-pair formation.^1−4^ PECs exhibit sensitivity to solution conditions such as pH, temperature, and ionic strength, making them highly responsive to external stimulus.^5−12^ They have garnered significant attention in biotechnology and materials science due to their potential applications in drug delivery,^13^ tissue engineering,^14^ coatings,^15^ batteries,^16^ and wearable sensors.^17,18^ Understanding the thermal behavior of PECs, particularly the glass transition temperature (*T*_g_), is essential for elucidating bulk physical properties of PECs.^19−24^ However, little focus has been placed on the effect of solvents besides water.

PECs typically assume a glassy state with extremely hard and brittle properties when dried, as reported by Michaels and co-workers in the 1960s.^25,26^ However, PECs are very sensitive to water absorption and become progressively more rubberlike and swollen. PECs can display a glass transition in the presence of water, and salt can further soften the complexes.^2,19,26−30^ In our previous studies of poly(allylamine hydrochloride) (PAH) and poly(acrylic acid) (PAA) complexes, the effect of water content on their glass transition behavior was examined using modulated differential scanning calorimetry (MDSC), for which dried PAH/PAA complexes did not show a detectable *T*_g_.^31^ With increasing water content, the *T*_g_ decreased due to water’s participation in the hydrogen-bonding network and its plasticization properties. The effects of salt and doping of the PEC have also been considered.^2,21,32^ Depending on the salt type, there is a general decrease in *T*_g_ as the doping level increases. Because added salt can break intrinsic ion pairs and form extrinsic ion pairs within the polyelectrolyte assemblies.^1,2,33,34^ These observations are summarized in a scaling^2,31^ by eq 1:
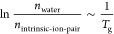
1where *n*_water_ and *n*_intrinsic-ion-pair_ are the respective moles of water molecules and intrinsic ion pairs in the same volume of PEC.

Given that water strongly effects the glass transition of PECs, it is necessary to clarify the microenvironment of water within polyelectrolyte assemblies.^2,7,31,35−37^ In hydrated PDADMA/PSS (poly(diallyldimethylammonium/poly(styrenesulfonate)) complexes, water mobility was characterized via all-atom detail molecular dynamics (MD) simulations and MDSC.^7^ The results illustrated that (1) at low hydration, only nonfreezing water was present, with water effectively immobilized around PE charge groups due to strong PE-water interactions, and (2) at high hydration, small amounts of freezing bound water emerged, with water possessing less tight bonding with PEs. Most PECs are nonstoichiometric, and additional counterions are required to balance the excess charges on PEs, leading to the generation of extrinsic ion pairs. In this case, water uptake caused by excess PE charge groups can result in higher free volume and chain mobility in PECs, leading to lower *T*_g_’s and moduli.^19^ Similarly, polyelectrolyte assembly mechanical properties and relaxation times are sensitive to bound water at the intrinsic ion pair and highly dependent on relative humidity.^20,23,38−41^

In contrast to water, organic solvents added to PECs can have varying effects. The Zacharia group^42,43^ demonstrated the contraction of PE multilayers upon exposure to various polar organic solvents. They discussed that the multilayers rigidified in organic solvents due to the decreased dielectric constant and increased hydrophobic interactions. The presence of the organic solvents (such as acetone, dioxane, or ethylene glycol monoethyl ether) was considered to compete with the PE’s proton-accepting groups for hydrogen bonding sites and disrupt PE–PE interactions.^44,45^ The effect of organic solvent on hydrogen bonds and PE conformation was also studied using MD simulations of a PAA chain in water/ethanol mixtures.^46^ With an increasing ethanol fraction, the un-ionized PAA chain expanded due to the creation of PAA-ethanol hydrogen bonds and the displacement of PAA-water pairs; however, the ionized PAA chain shrank with increasing ethanol content due to the enhanced interactions between COO^–^ groups and counterions. The Tirrell group^47^ found that salt resistance of PECs could be decreased by introducing cosolvents (ethylene glycol and ethanol), which facilitated the transition from two phases to a single phase at lower salt levels. Specifically, the relatively low polarity of ethanol compared with that of ethylene glycol formed a more “hydrophobic” environment, resulting in further reduced salt resistance of the PECs. In contrast, alcohols added to relatively hydrophilic polypeptide-based or polysaccharide-based complexes, led to increased PEC salt resistance, resulting in an opposite phase transition tendency.^48,49^ Our recent work indicated that added ethanol increased counterion condensation which resulted in a “looser” network, whereas urea readily formed a solvation shell and induced PE–PE separation due to its ability to replace water at the PSS charge group.^50^

In addition to the above, the effect of solvents on PEC mechanical and rheological properties has also been reported, but thermal transition studies are limited.^31,51−53^ Prior studies revealed that the addition of alcohols with low dielectric constants can reduce the degree of ionization of polyelectrolytes and enhance the mechanical properties by influencing the electrostatic effects in chitosan-based polyelectrolyte complexes.^51,52^ Schlenoff and co-workers reported a reduction in the *T*_g_ of PDADMA/PSS PECs when formamide is used instead of water as solvent.^54^ Altogether, the effects of organic solvents on PEC behavior have been analyzed in terms of hydrogen bonding effectiveness, hydrophobic interactions, and solvent polarity.

**Figure 1:**
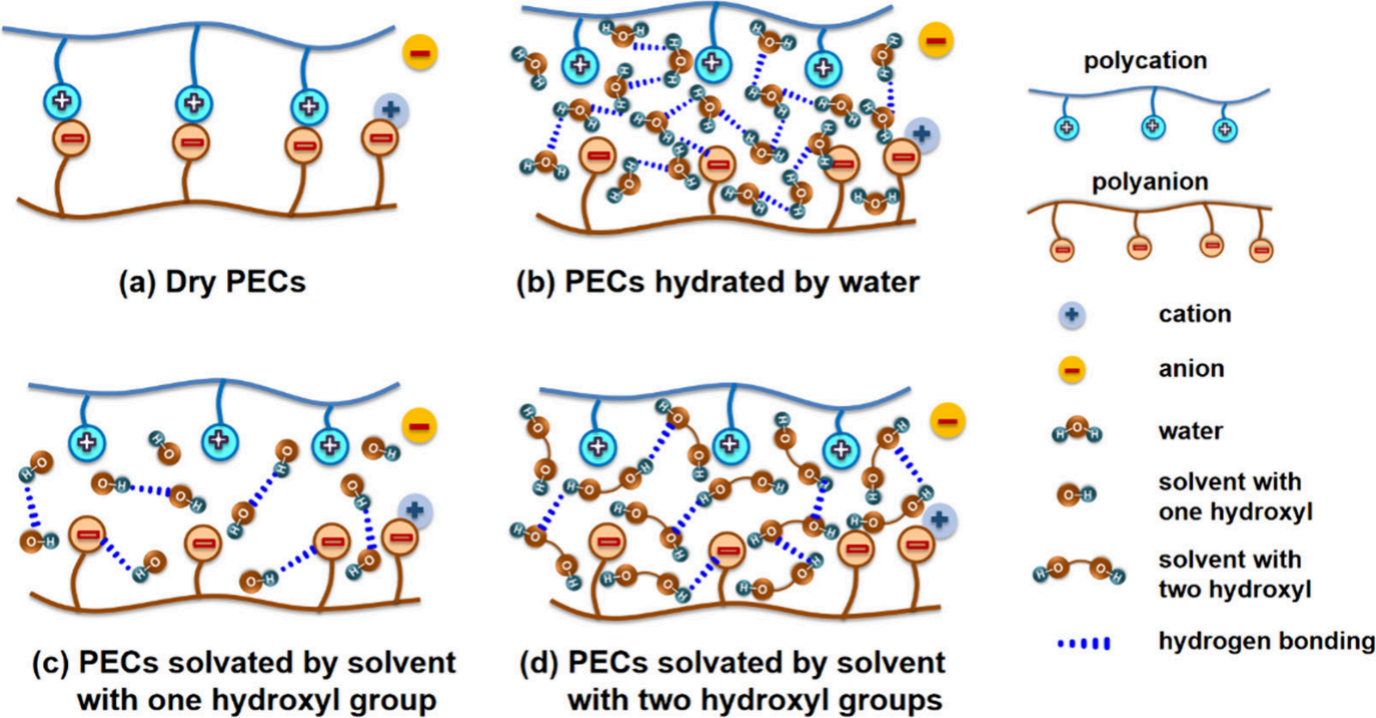
Schematic of polycation-polyanion interactions in (a) dry PECs, (b) PECs hydrated by water, (c) PECs solvated by a solvent with one hydroxyl group, and (d) PECs solvated by a solvent with two hydroxyl groups.

In the present work, we investigate the influence of varying solvents on the *T*_g_ of PDADMA/PAA complexes. This PEC system is selected because we hypothesized that the carboxylic acid group of PAA may hydrogen bond to varying degrees with the added solvent, thus modifying the *T*_g_. The selected solvents have varying numbers of hydroxyl groups (Table S1), allowing for a comparison with water. Figure 1 compares the possible hydrogen bonding networks that might arise for solvents with one or two hydroxyl groups or with water as the solvent. For dry PECs, intrinsic ion pairs formed between polycations and polyanions and extrinsic ion pairs between PEs and counterions participate are present; see Figure 1a. According to our prior reports,^23,31,55,56^ additional water can insert into the hydration shell and allow the assembly to relax via rearrangement of the intrinsic ion pairs with other adjacent intrinsic ion pairs or the formation of an extrinsic ion pair and disruption of the intrinsic ion pair, Figure 1(b). We hypothesize that monoalcohols and dialcohols might behave similarly (Figure 1c and d). MDSC was used to determine the glass transition behavior of the PECs in various solvents. The composition and ionization were analyzed using proton nuclear magnetic resonance (^1^H NMR) spectroscopy and attenuated total reflectance Fourier transform infrared (ATR–FTIR) spectroscopy. For molecular details of the interactions within the solvated PECs, all-atom molecular dynamics (MD) simulations were performed. These results are discussed within the context of eq 1 and its modification for solvents with hydrogen bonding interactions.

## Materials and Methods

### Materials

PAA (Mw = 100 000 g/mol, 35 wt % aqueous solution) and PDADMA (Mw = 200,000–350 000 g/mol, 20 wt % in H_2_O) were purchased from Sigma-Aldrich. The structures are shown in Figure S1. The PEs were used as received. Milli-Q water with a resistivity of 18.2 MΩ·cm was used to dissolve individual PEs and to prepare mixture solutions of PECs. Solvents included methanol (≥99.8%, purchased from J.T.Baker), ethanol (anhydrous/denatured, purchased from Beantown Chemical), ethylene glycol (≥99%, purchased from VWR Chemicals BDH), 1-propanol (≥99.5%, purchased from Alfa Aesar), 1,3-propanediol (98%, purchased from Sigma-Aldrich) and glycerol (≥99%, purchased from Sigma-Aldrich), respectively. Their structures are shown in Figure S2.

### Preparation of PECs

Liquid-like PDADMA/PAA complex coacervates were prepared under stoichiometric mixing conditions at pH 7.0 (Figure S3). The concentrations of PDADMA and PAA solutions were 100 mM with respect to the polymer repeat unit. The pH values of individual PE solutions were adjusted by using NaOH aqueous solutions. PDADMA solution (100 mL) was quickly added to 100 mL of PAA solution while stirring. The mixture was stirred at 600 rpm for 30 min. The mixture was centrifuged at 10000 rpm for 10 min, and the liquid coacervate was then separated and oven-dried without vacuum at 343 K overnight. The dried PECs were ground into a powder, vacuum-dried further at 393 K for 6 h at least, and then stored in a desiccator until further use. In addition, these fully dried PECs were later solvated with varying solvents at a certain content. For instance, 2.4 mg of ethanol was added to 7.6 mg of dry PEC to achieve a 24 wt % ethanol-solvated PEC.

### Modulated Differential Scanning Calorimetry (MDSC)

The *T*_g_ of PECs with various amounts and types of solvents was determined using MDSC (Q200, TA Instruments). Solvated PECs were prepared in hermetically sealed pans with lids and allowed to equilibrate for 1–2 weeks before measurement. The solvated PECs went through at least two cooling and heating cycles at 2 K/min, ramped from 278 to 383 K, except glycerol (from 295 to 383 K), with an amplitude of 1.272 K and a period of 60 s. The second heating cycle was used to determine the *T*_g_ values. The thermograms are shown in “exotherm down” format and correspond to the second heating cycle.

### Fourier Transform Infrared (FTIR) Spectroscopy

Attenuated total reflectance Fourier transform infrared (ATR–FTIR) spectroscopy (Jasco, FT/IR 4600) using a Quest diamond single-bounce ATR attachment (Specac) was used to calculate the degree of dissociation of PAA in the PECs. Spectra were collected by averaging 64 scans over a range of 600 cm^–1^ up to 4000 cm^–1^ at a resolution of 2 cm^–1^. Dried or hydrated with 24 wt % anhydrous ethanol PECs were used for the measurements.

### Proton Nuclear Magnetic Resonance Spectroscopy

Proton nuclear magnetic resonance (^1^H NMR) spectroscopy (400 MHz proton frequency, Avance Neo 400 spectrometer) was used to measure the composition of the PDADMA/PAA complexes. Dried complex (∼10 mg) was dissolved in 1 mL of 2.5 M KBr deuterium oxide (D_2_O). Then, the mixture was added to the NMR tube just prior to recording the spectra. The same approach was used for PDADMA and PAA, respectively.

### Number of Intrinsic Ion Pairs

The calculation of the number of intrinsic ion pairs was based on the following assumptions: (1) The mol % of PAA and PDADMA was that obtained using ^1^H NMR spectroscopy, and the degree of dissociation of PAA was that obtained using FTIR spectroscopy; (2) PDADMA was assumed to be fully charged at pH 7.0; and (3) PDADMA was assumed to only be involved in the formation of intrinsic ion pairs.

The number of intrinsic ion pairs in a PEC requires the calculation of the molar mass of the PEC. To calculate the molar mass of a PEC, consider a basis of 1 mol of repeat units. For PDADMA-PAA PECs prepared at pH 7.0, from ^1^H NMR spectroscopy, the PAA mol % (by repeat unit) = 57%. Thus, 1 mol of repeat units in a PDADMA-PAA PEC prepared at pH 7.0 contains 0.57 mol of PAA repeat units and 0.43 mol of PDADMA repeat units. Based on the above-mentioned assumptions and bases:

(1) Here, 0.43 mol of PDADMA is involved in the formation of intrinsic ion pairs with PAA. Thus, 0.43 mol of PAA is involved in formation of intrinsic ion pairs.
(2) The degree of dissociation of PAA in PDADMA-PAA PECs prepared at pH 7.0 is equal to 92%. In other words, the fraction of uncharged PAA is 8%. Thus, the remaining 0.0944 (=0.57*0.92–0.43) moles of PAA forms an extrinsic ion pair with sodium to maintain charge neutrality; and 0.0456 (=0.57*0.08) moles of PAA is nonionized state containing the hydroxyl group.

Figure S4 shows an intrinsic ion pair, an extrinsic ion pair, and an uncharged PAA. Based on the above discussion, 1 mol of repeat units in a PDADMA-PAA PEC is composed of 0.43 mol (repeat unit) of intrinsic ion pairs between PDADMA^+^ and PAA^–^, 0.0944 mol (repeat unit) of extrinsic ion pairs between PAA^–^ and Na^+^, and 0.0456 mol (repeat unit) of uncharged PAA. Therefore, the composite molar mass for PDADMA-PAA PEC is
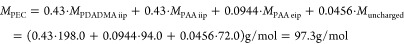
where *M*_PDADMA iip_ is the molar mass of PDADMA in an intrinsic ion pair, *M*_PAA iip_ is molar mass of PAA in an intrinsic ion pair, *M*_PAA eip_ is molar mass of PAA in an extrinsic ion pair and *M*_uncharged_ is the molar mass of uncharged PAA. More details are available in Supporting Information.

### Molecular Dynamics (MD) Simulations

**Figure 2:**
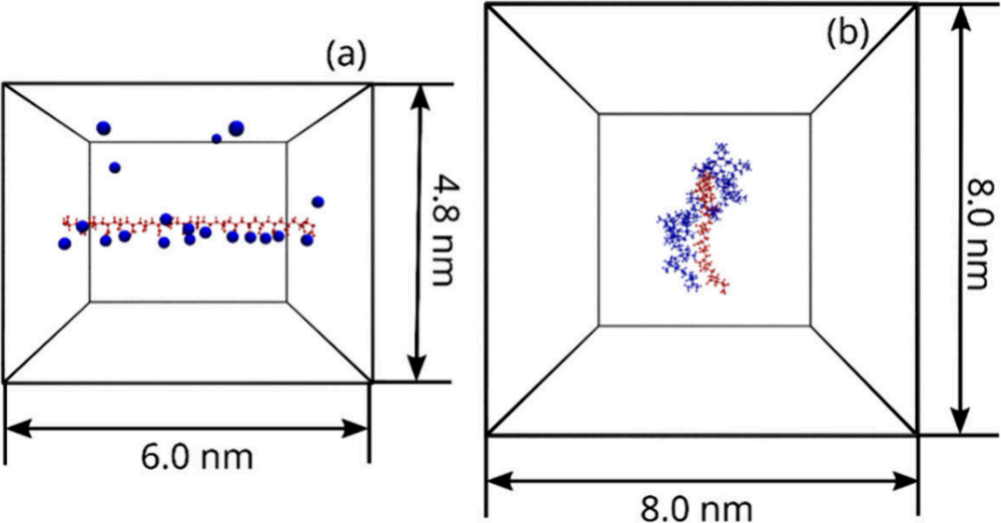
Simulation snapshot visualizations corresponding to the two sets of simulations performed. In (a), the 20 repeat units-long single PAA chain and its counterions are visualized. The PAA chain spans the simulation box axially as an infinite chain. A simulation box of 4.8 × 4.8 × 6.0 nm^3^ in size is considered. In (b), the PDADMA-PAA PEC model with both PEs (20 repeat units each) in length is visualized. A cubic simulation box (8.0 nm)^3^ is considered. The PAA chain is in red, and the PDADMA and Na^+^ ions are in blue. The solvent is omitted in the visualization for clarity. The visualizations show a configuration from the end of the simulation setups and correspond to a relaxed, representative snapshot.

The molecular details of PEC solvation were examined using all-atom MD simulations with water, methanol, ethanol, 1-propanol, ethylene glycol, and 1,3-propanediol as the solvents. The solvents were described in explicit atomistic detail. Glycerol was not considered in the simulations due to its extremely high viscosity, which results in prohibitively slow self-diffusion in atomistic detail simulation time scales. PAA and PDADMA chains of 20 repeat units were considered. For simplicity, the ionization degree of PAA was set as 100%, corresponding to the complete ionization and modeling best the ionization degree of PAA in the solvated PEC in our experiments (see the results section). Two different simulation sets were considered: in the first, a single PAA chain spanned the simulation box axially as a straight, infinite chain, and in the second, a complex formed by PDADMA and PAA chains was modeled. The single PAA chain system charge was neutralized by 20 Na^+^ counterions. Because the PEC formed by the PDADMA and PAA chains in the other system is charge neutral, no counterions were added to simplify that setup. The single PAA chain simulations were performed to model the complete solvation saturation of PAA in the PEC while the second set was to model the interaction between the PEs in different solvents. The simulated systems are visualized in Figure 2.

Interactions in the systems were modeled by the OPLS-AA force-field,^57^ with an extension for the ammonium group.^58^ The OPLS-AA compatible explicit TIP3P water model was used to model water as the solvent.^59^ The model and modeling setup choices, including the force-field, have been used by us also in our previous studies.^9,34,50,60−62^ The preparation of the infinite, axially straight PE chain is detailed in refs (50, 63).

For the simulations, the Gromacs 2024.1 software was used.^64,65^ The production run was performed at 300 K temperature maintained by the V-rescale thermostat with a time constant of 0.1 ps.^66^ The pressure was maintained at 100 kPa by the C-rescale isotropic barostat, with a time constant of 0.4 ps.^67^ Electrostatic interactions were treated with the particle-mesh Ewald method.^68,69^ Both for the real-space part of the electrostatics and the Lennard–Jones interactions, a cutoff distance of 1.0 nm was used. The P-LINCS algorithm was used to constrain the bond lengths of the hydrogen atoms.^64,65^ The integration time step was 1 fs. The simulations were 100 ns in duration, out of which 50 ns were used for data analysis. Data analysis was performed using Gromacs tools and Python scripts.^64,65^ For visualization of the MD trajectories, VMD was used.^70^

The simulation protocol and MD trajectories analysis details are provided in SI.

## Results and Discussion

### Modulated DSC of Solvated PECs

**Figure 3:**
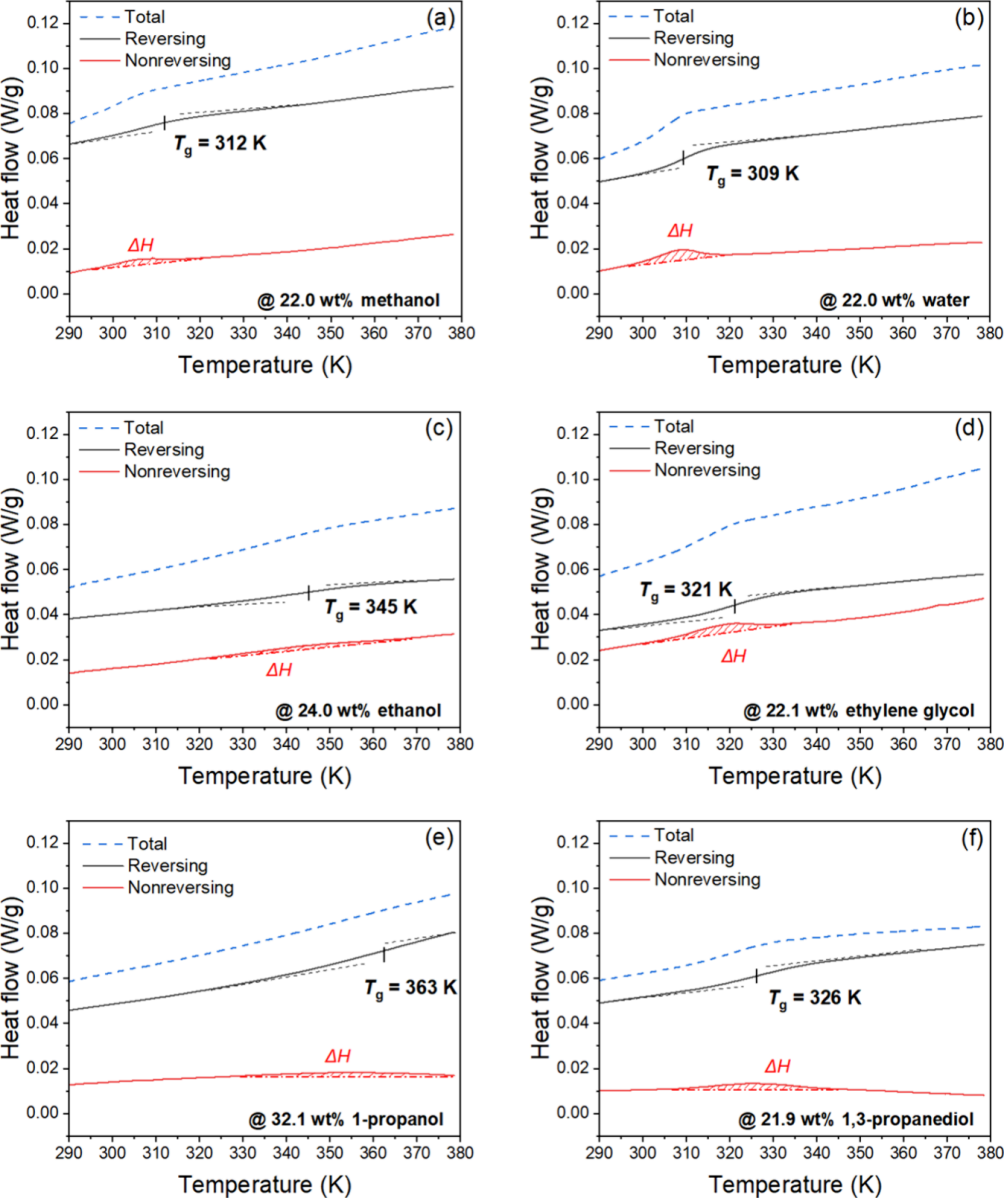
Representative MDSC thermograms of PDADMA/PAA complexes (no added salt) solvated with (a) 22.0 wt % methanol, (b) 22.0 wt % water, (c) 24.3 wt % ethanol, (d) 22.1 wt % ethylene glycol, (e) 32.1 wt % 1-propanol, and (f) 21.9 wt % 1,3-propanediol. Second heating scans were shown here with “exotherm down” format. Heating at 2 K/min, amplitude of 1.272 K for a period of 60 s.

Various alcohols were added to dried PDADMA/PAA PECs to assess the effects of alcohol quantity and type on the PEC’s *T*_g_ using MDSC. This method separates the reversing and nonreversing components of the heat flow curve. The reversing and non-reversing heat flows respectively contain information about reversible processes (such as the *T*_g_) and nonreversible processes (such as enthalpic relaxation, curing, *etc*.). For all of the solvents investigated, there is an obvious glass transition, characterized by the sigmoidal region in the reversing heat flow curve and the enthalpic relaxation in the nonreversing heat flow, Figure 3. Interestingly, PECs with solvents bearing one hydroxyl group (methanol, ethanol and 1-propanol in Figure 3a,c,e) show a smaller enthalpic relaxation in the nonreversing heat flow curve as compared with the cases of solvents with two hydroxyl groups (water, ethylene glycol and 1,3-propanediol in Figure 3b,d,f). In contrast, dried individual PEs and dried PDADMA/PAA PECs (with no solvents added) exhibited no detectable glass transition in the same temperature range (280–380 K), as shown in Figure S5. For glycerol, which has three hydroxyl groups and a high melting point (∼291 K), no *T*_g_ was observed, as shown in Figure S6. These results confirm that in most cases solvents plasticize the PEC and lower the *T*_g_ to a detectable range.

We next discuss the results of Figures 3, S5, and S6. Regarding the smaller enthalpic relaxation for the PECs containing alcohols with one hydroxyl group, the lower number of hydrogen bonds in the system may explain the weaker enthalpic relaxations displayed in Figure 3. In other reports, the number of hydrogen bonds in a system increases as the number of OH groups per molecule increases.^71,72^ As for the undetectable glass transition in the dry PEC of Figure S5, our previous work has shown that dried PECs and multilayers of other systems (PDADMA/PSS, PAH/PAA) are also glassy.^2,31,53,73,74^ These studies showed that when the PECs are hydrated, *T*_g_ values could be observed. Last, it is curious that the PEC containing glycerol did not exhibit a glass transition (Figure S6). One reason for this may be related to glycerol’s larger size and the strong hydrogen bond network forming within the solvent, which makes it more challenging to plasticize the PEC by inserting between polycation-polyanion intrinsic ion pairs.

### Comparing the Effects of Solvent Content and Number of Hydroxyl Groups on the PEC *T*_g_

**Figure 4:**
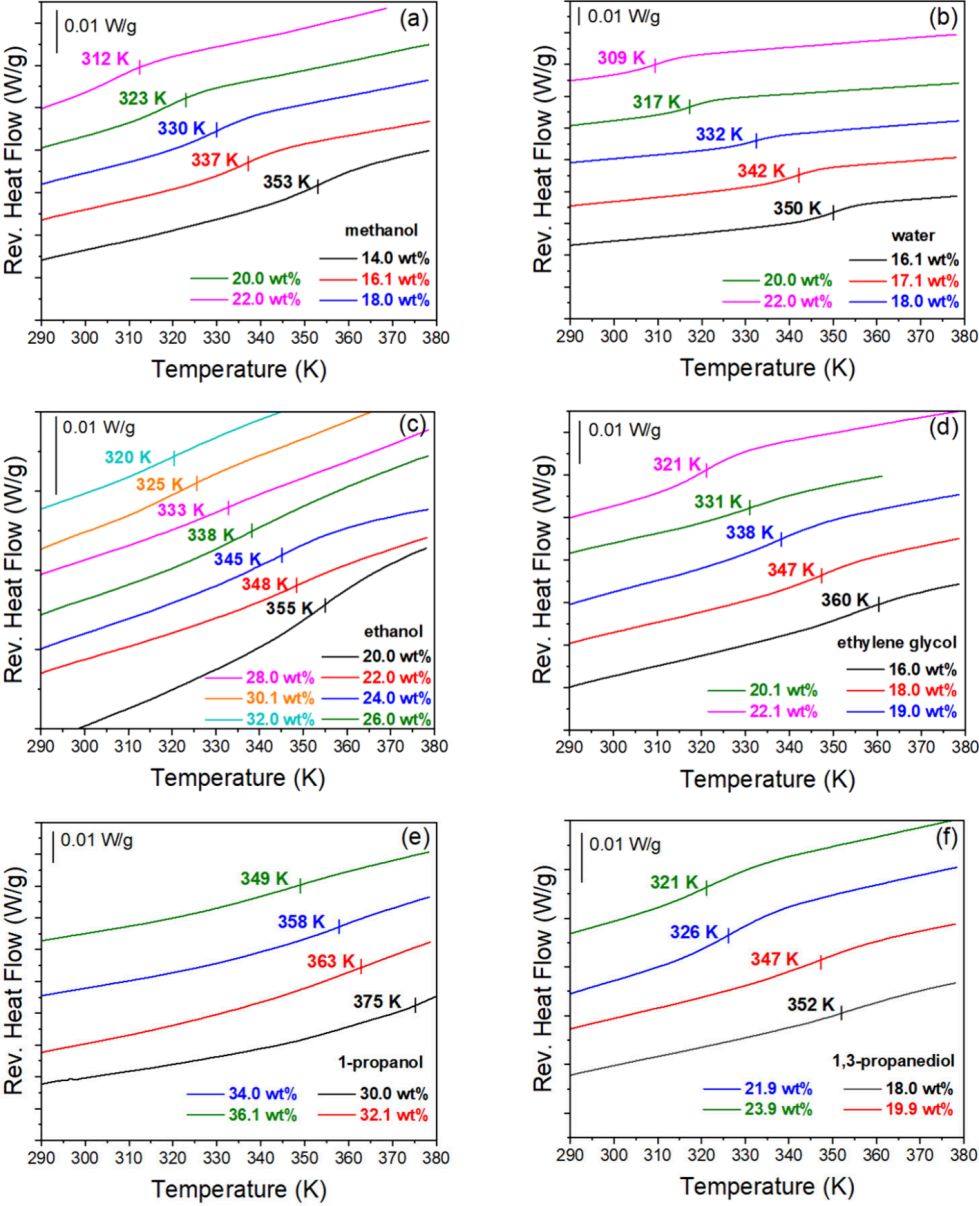
Reversing heat flows of PDADMA/PAA complexes for varying (a) the methanol content, (b) the water content, (c) the ethanol content, (d) the ethylene glycol content, (e) the 1-propanol content, and (f) the 1,3-propanediol content. Curves have been shifted along the *y*-axis for clarity. Reversing heat flow curves were taken from MDSC thermograms, similar to those described in Figure 2. The total and nonreversing curves were omitted for clarity.

The effects of varying solvent content on the PEC *T*_g_ were further examined using MDSC, Figure 4. Generally, *T*_g_ decreased with increasing solvent content. In the case of water, the PEC’s *T*_g_ decreased from 350 to 309 K as the water content increased from 16.1 to 22.0 wt %, Figure 4b. For PECs solvated by other alcohols with one (Figure 4a,c,e) or two (Figure 4d,f) hydroxyl groups, a similar trend in *T*_g_ was observed. As a plasticizer, water molecules can increase free volume, thereby promoting the movement of polymer chains by providing a lubrication effect.^20,30,31,75^ In addition, Zhang et al.^2^ reported that added water can decrease electrostatic attractions between polyelectrolyte intrinsic ion pairs. These prior findings provide insight into elucidating the role of alternative solvents, which may offer a similar mechanism of modifying the polyelectrolyte complex’s molecular environment.

**Figure 5:**
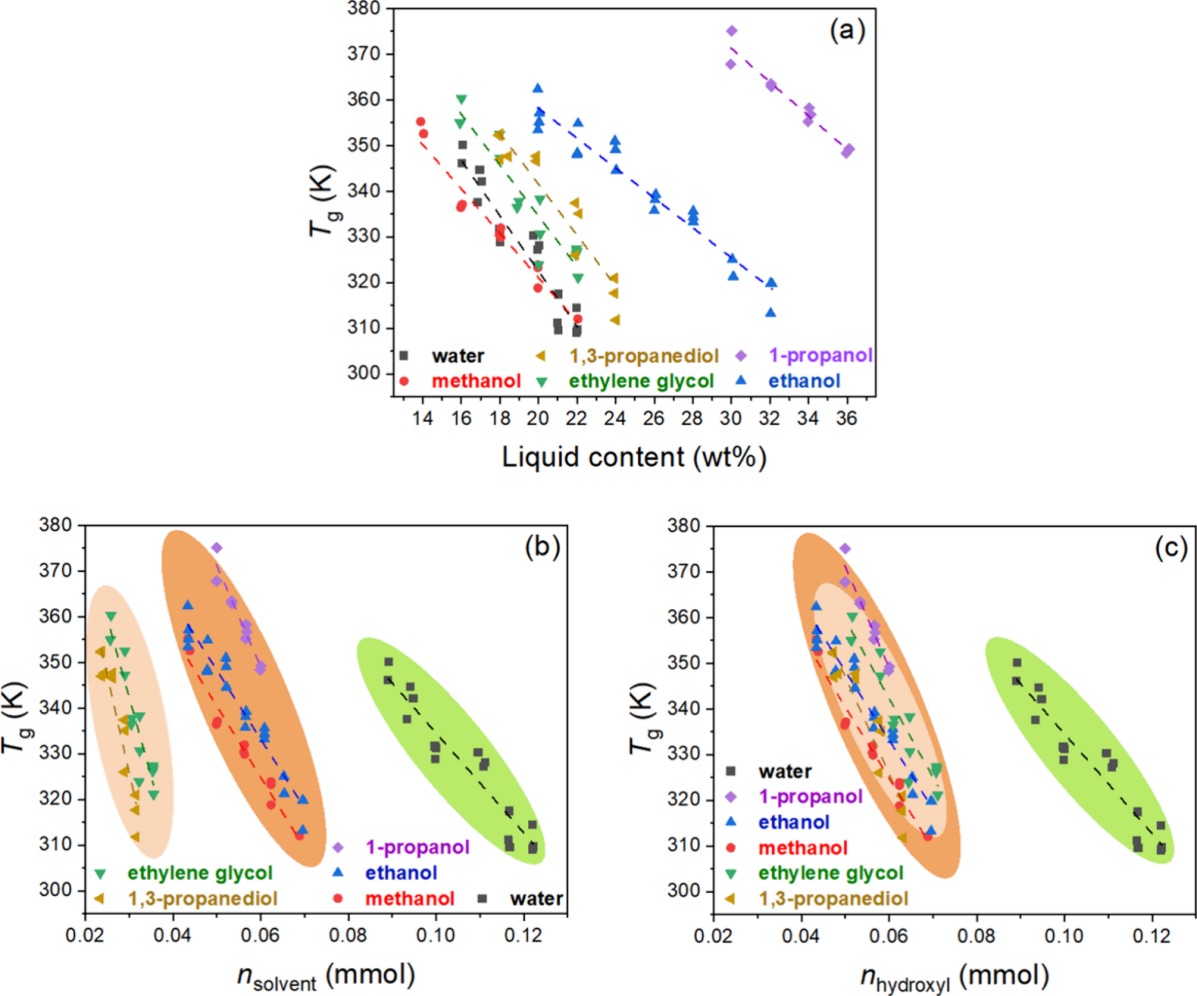
*T*_g_ for PDADMA/PAA complexes for solvents with varying (a) liquid content, (b) millimoles of solvent molecules (*n*_solvent_), and (c) millimoles of OH groups (*n*_hydroxyl_) available for solvation per 10 mg of solvated PEC (solvent + PEC mass, see Supporting Information). For OH group availability, diols were considered to have two available OH groups, monoalcohols, and the water molecule one available OH group.

To better compare the sensitivity of the *T*_g_ to the solvent content and the number of hydroxyl groups in that solvent, we plotted the PEC’s *T*_g_ versus the PEC’s liquid content, the moles of solvent molecules in the PEC, and the moles of hydroxyl groups from the solvent in the PEC. The curves for varying solvents show a similar general trend with increased solvent wt %, Figure 5a and Table S2. An increase of the solvent content leads to the solvation of PEs, promotes polymer chain relaxation, and decreases the PEC’s *T*_g_. We next examined the dependence of the PEC’s *T*_g_ on the amount of solvent added to complexes of similar mass such that the number density of the added solvent is expressed in millimoles “mmol” of solvent per 10 mg solvated PEC. Figure 5b and Table S3 show that, when plotted with the “mmol” of solvent (*n*_solvent_, using a basis of 10 mg solvated PEC), the *T*_g_-dependencies cluster into three groups classified as water, monoalcohols, and diols. Considering these groupings, the dependence of the *T*_g_ on the “mmol” of hydroxyl groups (*n*_hydroxyl_, one per molecule for monoalcohols and water (due to its small molecular sizes), two per molecule for diols) was examined. Remarkably, Figure 5c and Table S4 show that all of the *T*_g_ dependencies, except those corresponding to water as the solvent, collapse into one region. This result suggests that hydrogen bonding of the solvent with the PEC is involved in the glass transition process.

The molecular mechanism of the PEC’s glass transition with the addition of water has been well studied.^2,7,23,27,31,53,55,61,75^ As discussed above, water in the PEC reduces electrostatic interactions, disrupts intrinsic ion pairs,^61^ increases free volume, and acts as a lubricant for PE chains.^75^ Altogether, water enhances the PE motion, leading to a decrease in the *T*_g_. At low water concentrations, water molecules hydrate PEs and are strongly bound by charged groups.^7,75^ At a critical concentration, which corresponds to complete saturation of polymer hydration, an increase in water concentration leads to the appearance of relatively free water, which acts as a plasticizer, allowing melting/glass transition.^7,75^ We consider next that solvents with hydrogen-bonding groups may act in a similar way to water.

**Figure 6:**
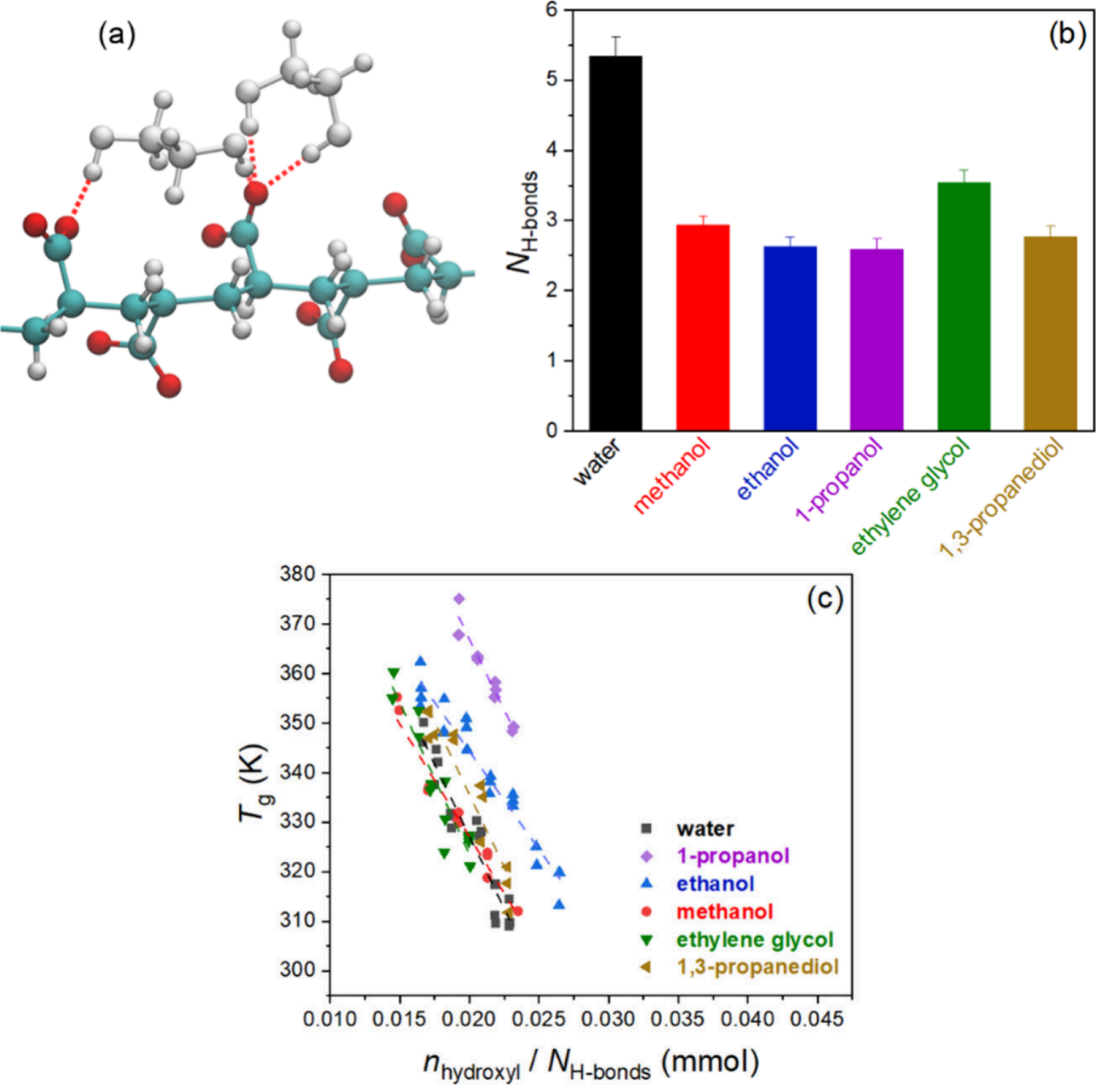
(a) Molecular level solvation of a single chain of PAA, illustrating the ability of the diols, here ethylene glycol, to form two hydrogen bonds with different carboxylate groups; (b) number of hydrogen bonds (*N*_H-bonds_) formed by the PAA carboxylate group in the MD simulations; (c) *T*_g_ dependence on the millimoles of solvent hydroxyl groups *n*_hydroxyl_ (Figure 5c) normalized by the number of hydrogen bonds *N*_H-bonds_ from (b). Analysis details are provided in SI.

Despite the collapse of the concentration values into the same region, except for water as the solvent, when the data was normalized by the number of OH groups, the curves show solvent-dependent variation. The observed variety of *T*_g_-dependencies for different solvents (shown in Figure 5c) can be explained by the difference in PE solvation in the studied systems. As the solvent’s OH groups appear crucial in the response, we focus on the PAA carboxylate groups as the key to solvation differences: hence, the solvation shell structure of fully charged PAA in the different solvents was studied via MD simulations. The simulations show that while monoalcohols form single hydrogen bonds between their OH group and the carboxylate groups of PAA, the diol solvent molecules can form hydrogen bonds with the carboxylate groups of PAA using both OH groups of the solvent (see Figure 6a). In consequence, for solvent molecules of comparable size, diols as solvents provide twice the number of hydrogen bond donors in comparison to the monoalcohols. The collapse of the *T*_g_ values to the same region, when normalizing by the OH group count for the alcohols, together with the simulation results, supports the conclusion of the solvation of the PAA chain playing a key role in the glass transition process. The outlying case of water, in principle capable of forming two hydrogen bonds, can be understood by the small molecular size of the water molecule. While the small size allows formation of a high number of hydrogen bonds from individual water molecules to PAA, increasing the amount of bound solvent molecules, a single water molecule, due to size restrictions, forms one hydrogen bond with PAA while the other donor site remains partially blocked due to the size constraint. Summarizing the findings, Figure 6b shows the number of hydrogen bonds formed by one PAA repeat unit in the simulations with the different solvents. Notably, the number of hydrogen bonds that a PAA unit can form in water is twice that of any of the examined alcohol solvents. The resulting higher saturation level of polymer solvation also shows an increased critical solvent molar concentration for this system (Figure 5c).

The finding that the hydrogen bonding between PAA and solvent indeed determines the critical solvent molar concentration for a glass transition becomes evident when the dependence of the *T*_g_ on the concentration of OH groups (Figure 5c) is normalized by the number of hydrogen bonds formed by one PAA carboxylate group, *N*_H-bonds_ (Figure 6b). Figure 6c plots the *T*_g_ against the ratio of OH groups stoichiometrically available from the solvent (*n*_hydroxyl_) to *N*_H-bonds_, for which good overlap is shown.

### The Degree of Ionization of PAA within the PEC and PEC Composition

**Figure 7:**
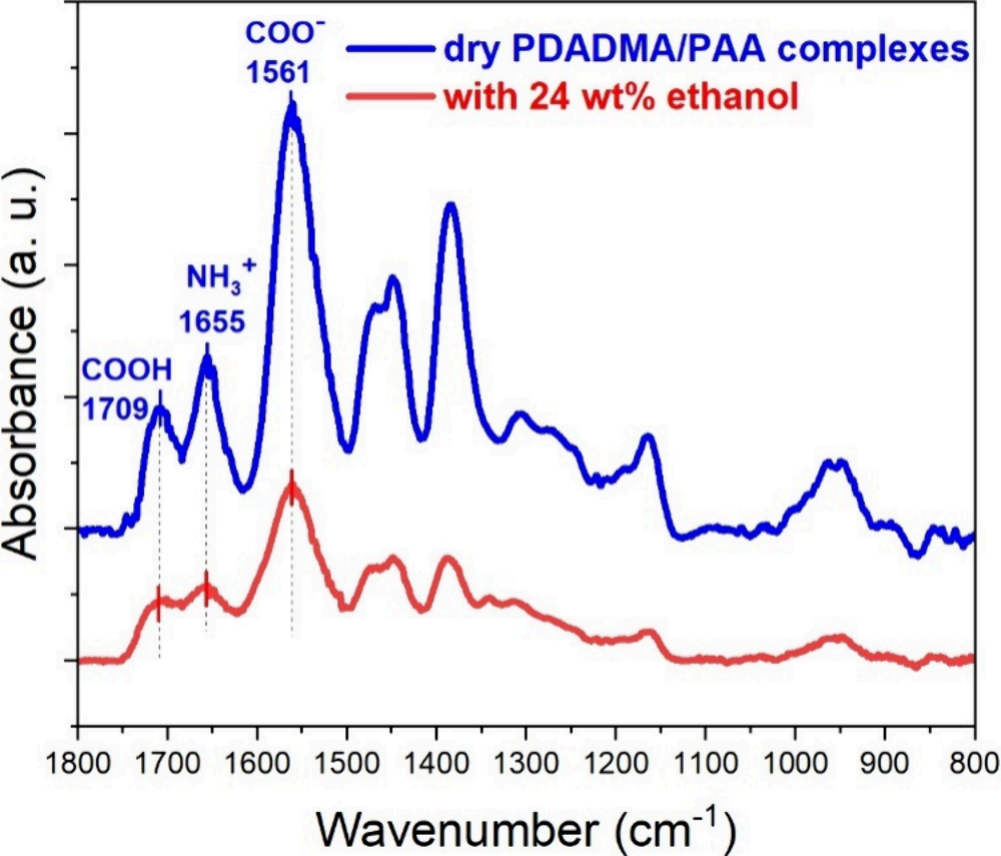
ATR-FTIR spectra for dried and solvated (24 wt % ethanol) PDADMA/PAA complexes prepared at pH 7 by mixing PDADMA and PAA aqueous solutions stoichiometrically.

To understand if the various solvents influence the ionization of PAA within the PEC, we compared the degree of ionization for a dried PDADMA/PAA PEC and one solvated with anhydrous ethanol (24 wt % ethanol in the PEC) using ATR–FTIR spectroscopy, Figure 7. The absorption band corresponding to the asymmetric stretching of −NH_3_^+^ (1655 cm^–1^) confirmed the presence of PDADMA. The absorption bands related to PAA’s carboxylic functional groups were observed at 1561 cm^–1^ (COO– asymmetric stretching) and 1709 cm^–1^ (C=O stretching in – COOH). We assumed that PDADMA was fully charged because it is a strong polyelectrolyte and that PAA could be partially ionized because it is a weak polyelectrolyte. The degree of ionization (α) of PAA within the complex was quantified using the following equation:^23,31^
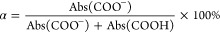
2where Abs (COO^–^) represents the absorbance of dissociated PAA carboxylate groups at 1561 cm^–1^ and Abs (COOH) is the absorbance of uncharged PAA at 1709 cm^–1^. The degree of ionization of PAA within the dried PECs prepared at pH 7.0 was 92%; in comparison, the degree of ionization of PAA within the PEC solvated with ethanol was 97%. This increase in ionization for PAA in the PEC with added ethanol is likely due to the decreased dielectric constant, which promotes ion pairing of the PAA and PDADMA charge units.

Next, ^1^H NMR spectroscopy was used to quantify the PEC’s composition, Figure S7. The PAA mol %, which represents the fraction of PAA repeat units in the dry PEC, was 57%; the PDADMA mol % was 43%. This indicates that the supernatant, which was discarded after centrifuging the PEC mixture, was likely rich in PDADMA (due to the asymmetry in the PEC’s composition).

### Scaling of the *T*_g_ with Solvent Hydroxyl Groups and Intrinsic Ion Pairing

**Figure 8:**
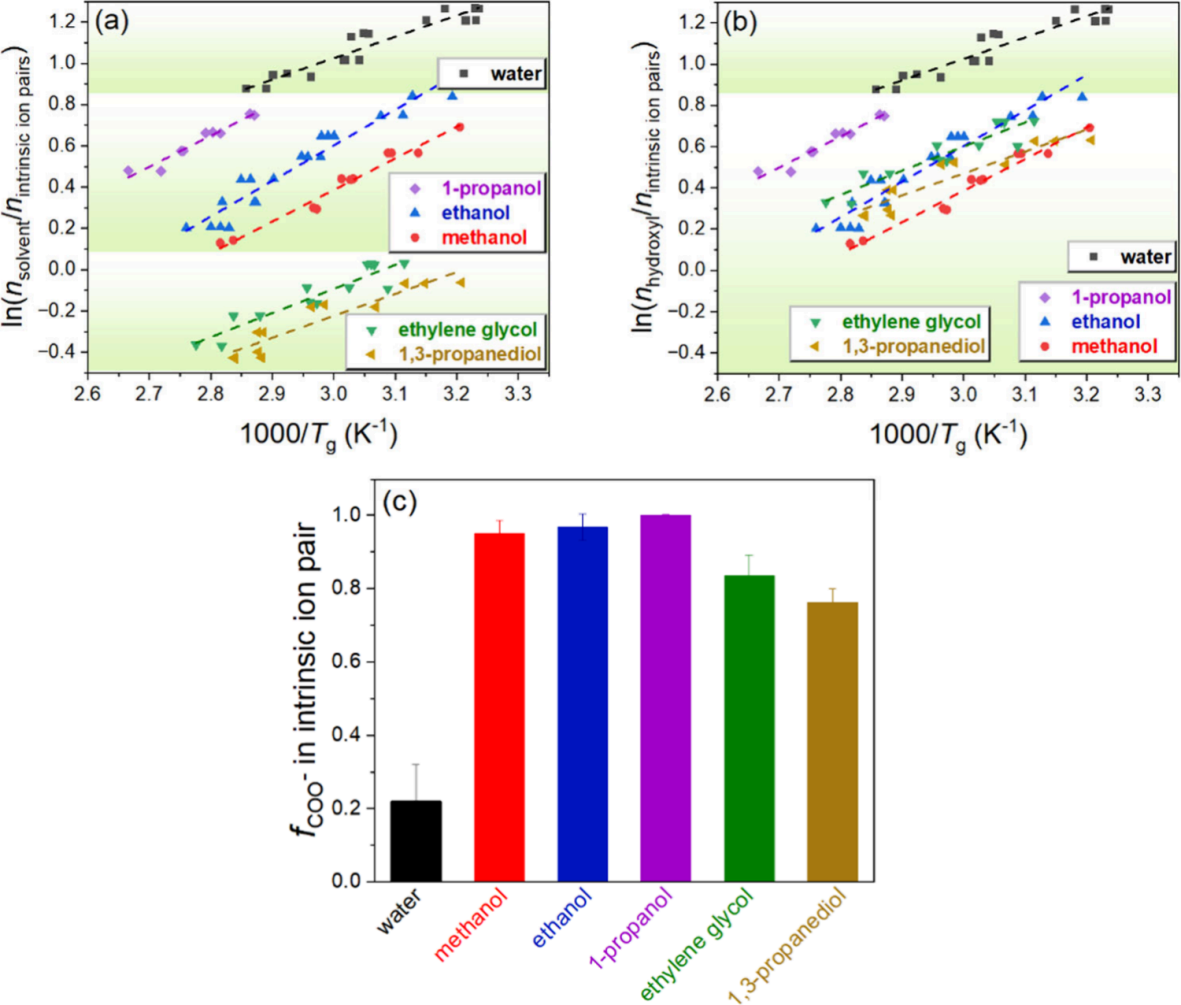
Linear fitting of (a) ln(*n*_solvent_/*n*_intrinsic-ion-pair_) vs 1000/*T*_g_ and (b) ln(*n*_hydroxyl_/*n*_intrinsic-ion-pair_) vs 1000/*T*_g_ for PDADMA/PAA complexes in varying solvents. (c) Fraction of carboxylate groups involved in intrinsic ion pairing in the MD simulations of PECs. Analysis details for the simulations are listed in SI.

To further explore the glass transition and its relationship to the mole ratio of solvent molecules to intrinsic ion pairs in the PDADMA/PAA complexes, we first plot the data in the form of  vs , shown in Figure 8a. *n*_intrinsic-ion-pair_ and *n*_solvent_ are the number of intrinsic ion pairs and added solvent, respectively; considering that both are in the same volume of complex, they can also be equivalent to a concentration. The calculation is explained in the Methods Section and in SI. Based on the van ’t Hoff relationship,^2,55^ we previously derived the following equation for PECs with added water in prior work: . Because the data in Figure 8a also follow the same linear form, we may apply this same relationship to the solvents. Here, *y* is the critical fraction of ion pairs that are solvent-separated when there is a bulk-scale relaxation of the PECs when *T* = *T*_g_. The van ’t Hoff enthalpy (Δ*H*) and entropy (Δ*S*) are associated with the transformation of the intrinsic ion pair from being a closely bound ion pair to becoming a more solvent-separated ion pair, which can be calculated from linear fits of the data, as listed in Table S5. The values of Δ*H* range from 8.3 to 14.1 kJ/mol for PECs for water, monoalcohols and diols; considering the standard deviation in these measurements, they all fall within error of the value of 10.5 ± 2.5 kJ/mol for the disruption of one O–H···O unit.^76^ This indicates that the energy associated with the glass transition can be affected by solvation due to hydrogen bonding.

To examine if the scaling should follow the hydroxyl concentration instead of the solvent concentration, we also plotted  vs in Figure 8b, where *n*_hydroxyl_ is the number of hydroxyl groups from the solvent molecules. The results show closer clustering of the data related to monoalcohols and diols, suggesting a stronger correlation between *T*_g_ and the number of hydroxyl groups for solvated PECs. The corresponding slopes and intercepts of Figure 8b are listed in Table S6, for which the slopes remained the same as compared to those in Figure 8a and Table S5.

The effect of solvents on the PDADMA-PAA interactions was examined via MD simulations of two PE chains (PDADMA and PAA) in different solvents. A clear correlation between the ability of the solvent to compete for the interaction with the PAA carboxylate groups (in the MD simulations) and the *T*_g_ values in the experiment was observed. Figure 8c demonstrates the fraction of PAA carboxylate groups involved in intrinsic ion pairing in the MD simulations of the two PEs chains. Figure 6b shows that for the monoalcohols a longer carbon chain in the solvent molecule decreases hydrogen bonding, which is accompanied by an increase in the fraction of carboxylate groups involved in intrinsic ion pairing in the MD simulation. The increase in intrinsic contacts follows the sequence methanol < ethanol < 1-propanol; The shifts in the *T*_g_ distributions (see Figure 8a,b) follow this same solvent quality order. For the diols, the effect is opposite, as the longer carbon chains provide additional conformational freedom for forming two hydrogen bonds by one solvent molecule. However, here the effect of the strength of the hydrogen bonds becomes evident in the data: Figure S8 demonstrates that 1,3-propanediol hydrogen bonds have a narrower distribution of angles than those of ethylene glycol which indicates that the hydrogen bonding in 1,3-propanediol is stronger.^77^ This leads to a smaller number of intrinsic ion pairs in 1,3-propanediol than in ethylene glycol. Furthermore, the smallest number of intrinsic ion pairs in the simulations is when water is the solvent, as the water hydrogen bond count is double to the other examined solvents (see Figure 6c). This is also shown in the *T*_g_ distribution shifting to lower temperatures in water.

## Conclusions

The effect of various solvents on the *T*_g_ of PDADMA/PAA complexes was investigated. For dry PECs, no detectable glass transition was observed, but one appeared upon the addition of different alcohols. This observation echoes our prior work with water, but here we considered the influence of the number of hydroxyl groups contributed by each solvent.

For monoalcohols with higher carbon content, the solvent quality decreased, and the PEC *T*_g_ increased. In the case of diols, those with a higher carbon content had additional flexibility to form stronger H-bonds, which increased the quality of the solvent and decreased the PEC’s *T*_g_. The largest number of hydrogen bonds was observed for water, which explains the lower *T*_g_ values for the PEC.

The linear relationship between the natural logarithm of ratio of the number of hydroxyl groups from the solvent molecules to intrinsic ion pairs () vs 1/*T*_g_ showed the influence of the solvent acting on the molecular relaxation at the intrinsic ion pair in PDADMA/PAA. This shows the influence of hydrogen bonding between the solvent and the intrinsic ion pair.

We demonstrated a clear correlation between the chemical structure of the solvent and its plasticization effect. MD simulations and the experimental data together reveal that the solvent effects arise from the differences in the interaction of the solvents with the PAA carboxylate group. For diols (1,3-propanediol, ethylene glycol), the glass transitions were twice as low compared with the monoalcohols (methanol, ethanol, 1-propanol) at equivalent concentrations. Meanwhile, water, which also has two hydrogen bond donors, is so small by its molecular size that its ability to form double hydrogen bonds with the carboxylate group was restricted. However, due to water’s small molecular size, more molecules can interact with PAA carboxylate groups. This allows for more effective solvation without disturbing intrinsic ion pairing.

These findings highlight the important role of the molecular interactions between PAA charge groups and solvent’s hydroxyl groups in the thermal properties of the PEC and provide a simple guideline for the prediction of the *T*_g_. Taken together, the impact of this work is that we have shown that alcohols by themselves can facilitate the glass transition in solvated PECS and we have explained this phenomenon at a molecular-level scale.

## Data Availability

Data associated with the molecular simulations of the work, including simulation input files, number data for the figures, and analysis scripts are available at https://doi.org/10.23729/236c360a-339d-495a-b79d-1050e6cbcdee.

## Supporting Information Available

Structures of PEs and PECs, optical microscopy images of complexes coacervate, MDSC results of dry PECs and the PECs solvated with anhydrous glycerol, ^1^H NMR spectroscopy results, and tables corresponding to slops/intercepts and the information on solvent we used; MD simulations protocol, additional details of simulations, and analysis details (PDF)
