# Supplementary material for: Solvent-Responsive Glass Transition Behavior of Polyelectrolyte Complexes
Source: Macromolecules. 2024 Dec 23;58(1):292–303. doi: 10.1021/acs.macromol.4c02417 (PMC11741136; doi:10.1021/acs.macromol.4c02417)
Supplement: Supplementary file 1 — ma4c02417_si_001.pdf [file ma4c02417_si_001.pdf]

Solvent-responsive glass transition behavior of polyelectrolyte complex

*Hongwei Li,[1] Dmitry Tolmachev,[2,3] Piotr Batys,[4] Maria Sammalkorpi,[2,3,5] Jodie L. Lutkenhaus[1,5]**

1. Artie McFerrin Department of Chemical Engineering, Texas A&M University, College Station, Texas 77843, United States

2. Department of Chemistry and Materials Science, Aalto University, P.O. Box 16100, 00076 Aalto, Finland

3. Academy of Finland Center of Excellence in Life-Inspired Hybrid Materials (LIBER), Aalto University, P.O. Box 16100, 00076 Aalto, Finland

4. Jerzy Haber Institute of Catalysis and Surface Chemistry, Polish Academy of Sciences, Niezapominajek 8, 30-239 Krakow, Poland

5. Department of Materials Science and Engineering, Texas A&M University, College Station, Texas 77840, USA

Corresponding email: jodie.lutkenhaus@tamu.edu

(a) PDADMA (b) PAA

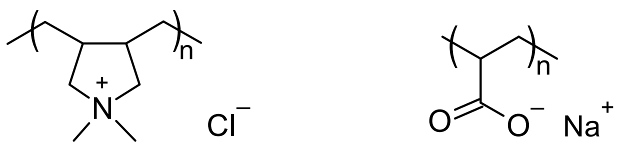

Figure S1. Chemical structures of (a) PDADMA and (b) PAA

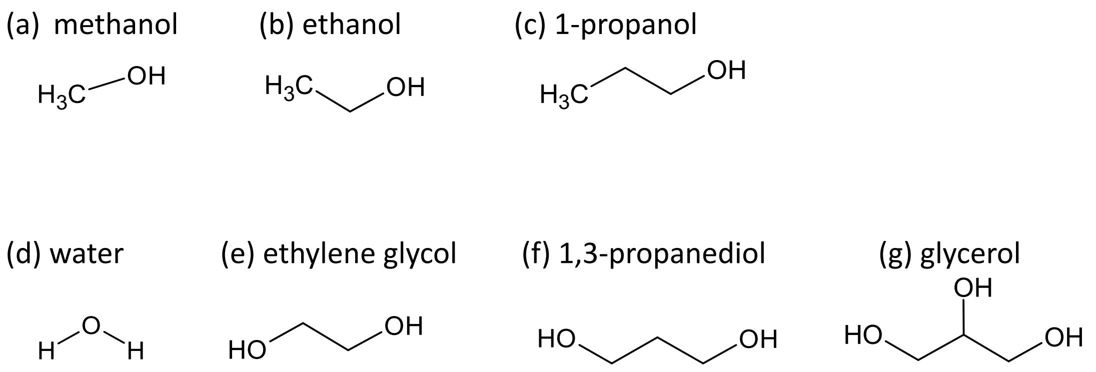

Figure S2. Chemical structures of (a) methanol, (b) ethanol, (c) 1-propanol, (d) water, (e) ethylene glycol, (f) 1,3-propanediol and (g) glycerol.

(a) (b)

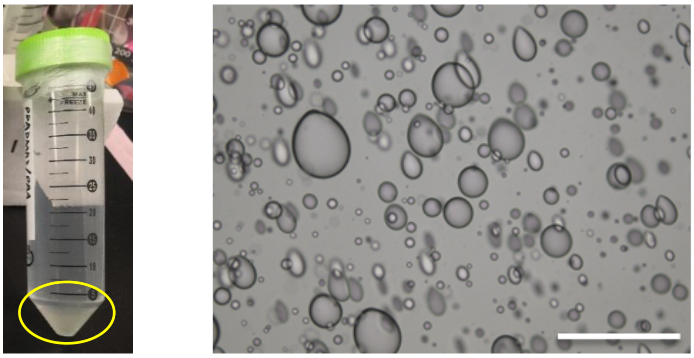

Figure S3. (a) Macroscopically separated phases after centrifuging and (b) optical microscopy images of coacervate droplet morphology of PDADMA/PAA complexes. Scale bar is 300 μm.

Calculation: Number of intrinsic ion pairs and number of solvent molecules

As a specific example, consider for PDADMA/PAA PECs prepared at pH 7.0 and hydration (W_{H_2O}) of 20 wt %, and a total mass of 100 g (water + PECs). In this case: Weight of water is 20 g and weight of PECs is 80 g.

Number of moles of water (n_{water}) = Weight of water/Molar mass of water = 20 g / (18.0 g/mol) = 1.11 mol

Numbers of hydroxyl groups of water ($n_{hydroxyl}$) = Number of moles of water (n_{water}) = 1.11 mol = 2.22 mol (Here, it is assumed that one hydroxyl group is contained in a water molecule.)

Number of moles of repeat units in a PEC = Weight of PEC/Molar mass of PEC = 80 g / (97.3 g/mol) = 0.82 mol

Besides, 1 mole of repeat units in the PEC have 0.43 moles of PDADMA occupied in intrinsic ion pairs, and every mole of PDADMA is equivalent to an intrinsic ion par. Thus, the number of moles of intrinsic ion pairs in 0.82 moles of repeat units in the PEC is:

Number of moles of intrinsic ion pairs ($n_{intrinsic\ ion\ pairs}$) = 0.82 mol*0.43 = 0.35 mol

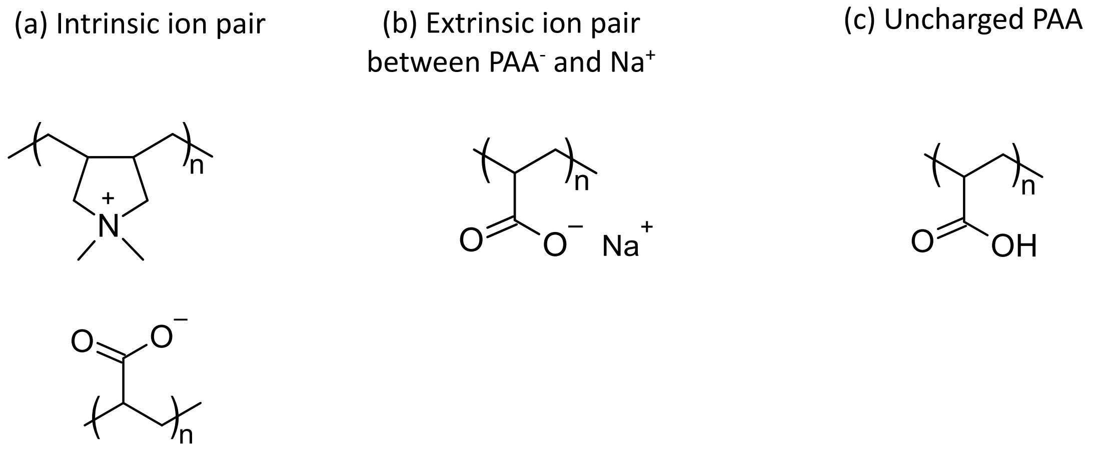

Figure S4. Repeat units and the corresponding molar mass of (a) Intrinsic ion pair between PDADMA$^+$ and PAA$^-$, (b) Extrinsic ion pair between PAA$^-$ and Na$^+$, and (c) uncharged PAA.

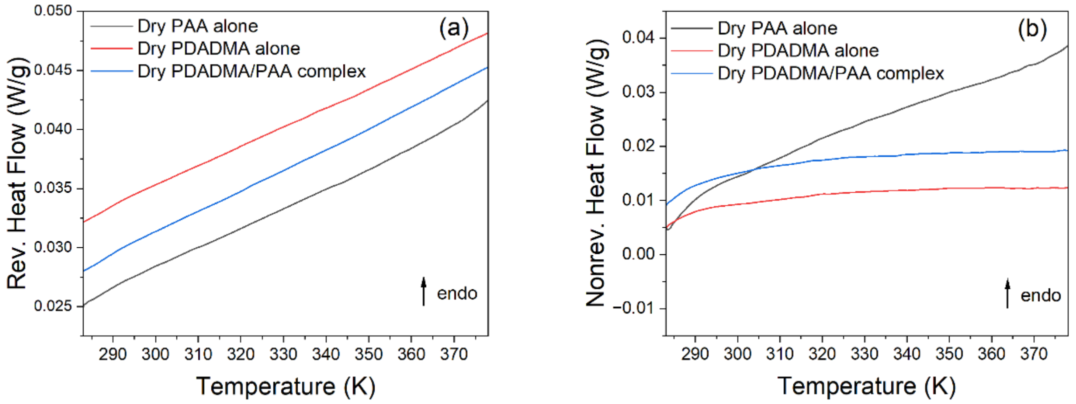

Figure S5. (a) Reversing and (b) nonreversing heat flows of dried PAA, PDADMA and PDADMAC/PAA complexes.

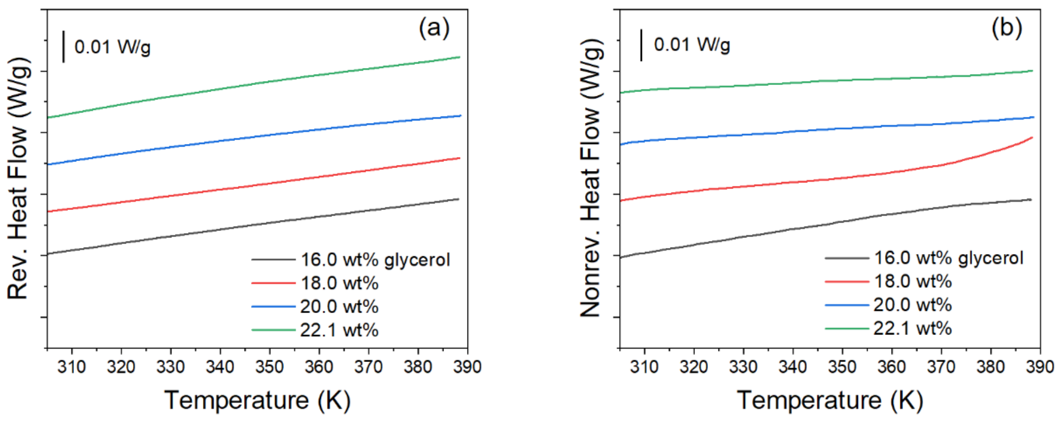

Figure S6. (a) Reversing and (b) nonreversing heat flows of PDADMAC/PAA complexes for varying glycerol content. Curves have been shifted along y-axis for clarity.

Table S1. Characteristic properties of the various solvents.

Solvent	Molar mass	Density	Melting point	Boiling point	Dielectric constant *	Hydroxyl number	Weight fraction of Hydroxyl
water	18.015 g/mol	0.997 g/ml	0.00 °C	99.98 °C	78.5	1	/
methanol	32.042 g/mol	0.792 g/ml	−97.60 °C	64.70 °C	32.6	1	53.08%
ethanol	46.069 g/mol	0.789 g/ml	−90.00 °C	78.00 °C	24.3	1	36.92%
ethylene glycol	62.068 g/mol	1.110 g/ml	−12.90 °C	197.30 °C	37.7	2	54.80%
1-propanol	60.096 g/mol	0.083 g/ml	−126.00 °C	97.00 °C	20.1	1	28.30%
1,3-propanediol	76.095 g/mol	1.060 g/ml	−27.00 °C	217.00 °C	35.0 (20℃)	2	44.70%
glycerol	92.094 g/mol	1.261 g/ml	17.80 °C	290.00 °C	42.5	3	55.40%

* The dielectric constant value is at 25℃ temperature, unless specified otherwise.[1]

Table S2. Intercepts and slopes corresponding to solvent vs T_g, Figure 5a.

	methanol	ethanol	1-propanol	ethylene glycol	1, 3-propanediol	water
Intercept	418 ± 6.2	423 ± 5.1	480 ± 13.6	440 ± 18.8	450 ± 15.9	440 ± 13.5
Slope	-4 ± 1.7	-3 ± 0.5	-3 ± 1.1	-5 ± 1.2	-5 ± 1.2	-6 ± 0.6

Table S3. Intercepts and slopes corresponding to 'mmol' of solvent vs T_g, Figure 5b.

	methanol	ethanol	1-propanol	ethylene glycol	1, 3-propanediol	water
Intercept	418 ± 6.2	423 ± 5.1	480 ± 13.6	440 ± 18.8	450 ± 15.9	440 ± 13.5
Slope	-1500 ± 165.4	-1500 ± 87.4	-2200 ± 228.9	-3400 ± 463.2	-4200 ± 509.4	-1000 ± 183.6

Table S4. Intercepts and slopes corresponding to 'mmol' of OH groups vs T_g, Figure 5c.

	methanol	ethanol	1-propanol	ethylene glycol	1, 3-propanediol	water
Intercept	418 ± 6.2	423 ± 5.1	480 ± 13.6	440 ± 18.8	450 ± 12.9	440 ± 13.5
Slope	-1500 ± 165.4	-1500 ± 87.4	-2200 ± 228.9	-1700 ± 231.6	-2100 ± 254.7	-1000 ± 183.6

Table S5. Intercepts and slopes corresponding to $ln\frac{n_{\text{solvent}}}{n_{\text{intrinsic ion pair}}}$ vs $\frac{1}{T_g}$, Figure 8a.

	methanol	ethanol	1-propanol	ethylene glycol	1, 3-propanediol	water
Intercept	-4.2 ± 0.3	-4.5 ± 0.4	-3.5 ± 0.5	-3.6 ± 0.4	-3.4 ± 0.4	-2.1 ± 0.3
Slope	1.5 ± 0.1	1.7 ± 0.1	1.5 ± 0.2	1.1 ± 0.2	1.0 ± 0.2	1.0 ± 0.1
ΔH (*kJ*/mol)	-12.5 ± 0.8	-14.1 ± 0.8	-12.5 ± 1.7	-9.1 ± 1.7	-8.3 ± 1.7	-8.3 ± 0.8

Table S6. Intercepts and slopes corresponding to $ln\frac{n_{\text{hydroxyl}}}{n_{\text{intrinsic ion pair}}}$ vs $\frac{1}{T_g}$, Figure 8b.

	methanol	ethanol	1-propanol	ethylene glycol	1, 3-propanediol	water
Intercept	-4.2 ± 0.3	-4.5 ± 0.4	-3.5 ± 0.5	-2.9 ± 0.4	-2.7 ± 0.4	-2.1 ± 0.3
Slope	1.5 ± 0.1	1.7 ± 0.1	1.5 ± 0.2	1.1 ± 0.2	1.0 ± 0.2	1.0 ± 0.1
ΔH (*kJ*/mol)	-12.5 ± 0.8	-14.1 ± 0.8	-12.5 ± 1.7	-9.1 ± 1.7	-8.3 ± 1.7	-8.3± 0.8

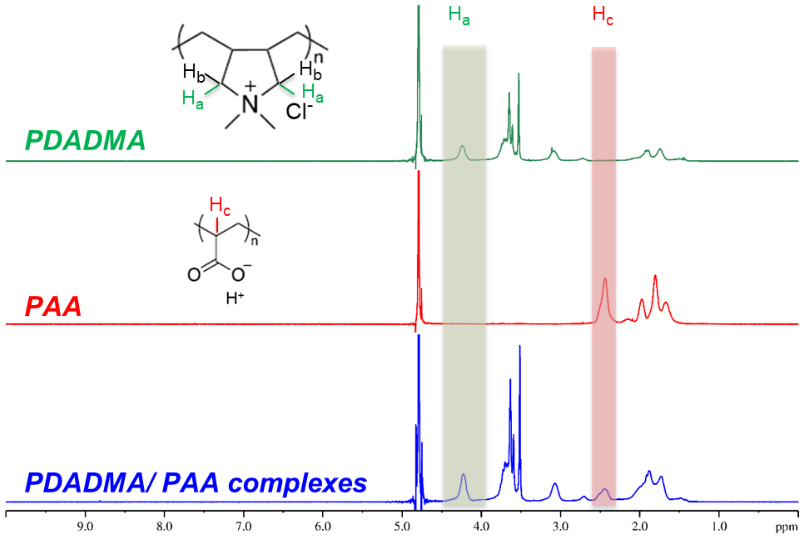

Figure S7. ^{1}H-NMR spectra for homopolymer PDADMA, homopolymer PAA, and PDADMA/PAA complexes.

Based on Figure S7, the fraction of PAA repeat units (PAA mol%) in the dry PEC was obtained using the equation below:

$$PAA\ mol\% = \frac{PAA}{PAA + PDADMA} = \frac{A(H_c)}{A(H_c) + \frac{1}{2}A(H_a)} = \frac{1}{1 + 0.75} = 57\%$$

Where $A(H_c)$ is the NMR area for the characteristic peak of carbon atom at the "c" position in PAA and $A(H_a)$ is the NMR area for the characteristic peak of carbon atom at the "a" position in PDADMAC, respectively.

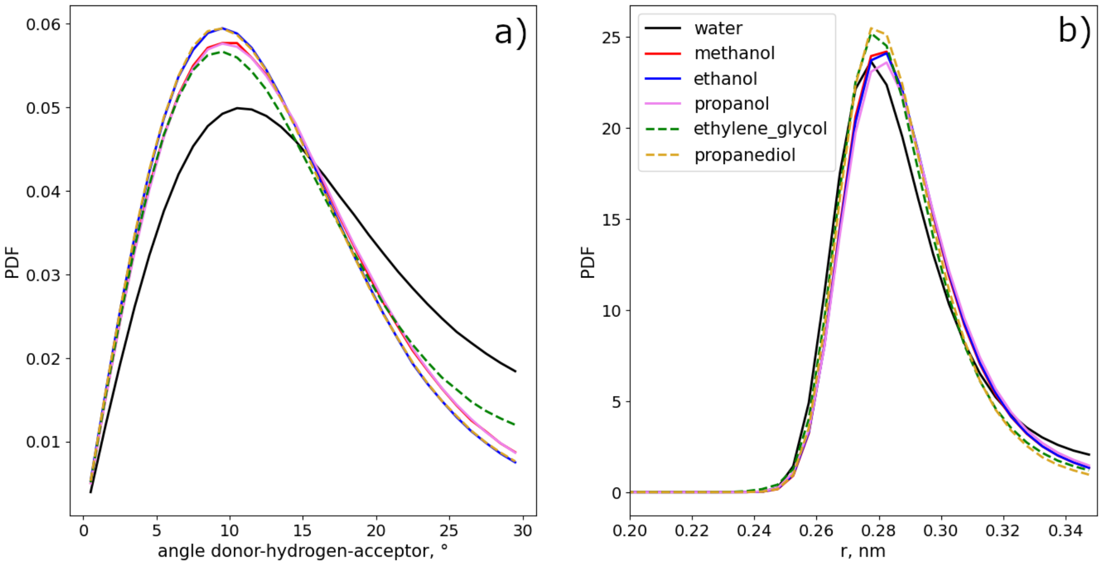

Figure S8. (a) The distance and (b) angle distribution of hydrogen bonds between the polyanion and different solvents.

MD simulations protocol

The preparation of a solvation box with pure solvent for the MD simulations:

1.1. The solvent molecules were packed into the simulation box by random placement to reach a close match with the experimental densities by the Packmol software.[2] As the box size, 4.0 x 4.0 x 4.0 nm^3 was used as target size.
1.2. The simulation system was energy minimized.
1.3. An NVT ensemble simulation for 2 ns was performed for initial relaxation. In this, temperature maintained at 300 K by the Berendsen thermostat[3] with time constant 0.1 ps.
1.4. An NPT ensemble simulation for 10 ns at 360 K and 100 kPa was performed for continuation of the initial relaxation. Temperature and pressure were maintained by the Berendsen thermostat and barostat,[3] time constants 0.1 ps and 0.2 ps respectively.
1.5. An NPT simulation for 10 ns at 300 K and 100 kPa was performed. Temperature and pressure maintained by Berendsen thermostat and barostat,[3] same parameters.

The preparation of the simulation boxes for the production run:

2.1. The models of the PEs (the PAA chain spanning the simulation box as an infinite chain, and the free-ended PAA and PDADMA chains) were created and used in our previous works.[4-10]

2.2. The solvent simulation box created in the pure solvent simulations was used to solvate the PE chains using the **gmx solvate** tool. For the single PE chain system, counterions were added by random replacement of solvent molecules by the ions.

2.3. The simulation system that included the solvated PE(s), and when present, ions, was energy minimized.
2.4. Initial relaxation was performed by an NVT ensemble simulation run for 50 ns at 360 K temperature. In this, the temperature was maintained by the V-rescale thermostat[11] using a time constant 0.1 ps.
2.5. The systems were equilibrated by an NPT ensemble simulations run for 50 ns at 300K. Temperature and pressure were maintained by the V-rescale thermostat[11] and C-rescale barostat[12], time constants 0.1 ps and 0.4 ps respectively. As a criterion for the equilibration of the system, the time evolution of the number of hydrogen bonds between solvent and PAA for single PAA chain simulation and the number of intrinsic ion pairs for polyelectrolyte complex simulation were used.
Finally, using the same protocol, the production runs were 50 ns. This time period was used for all simulation data analysis.

MD simulations analysis details.

An ion pair was defined as intrinsic (in the analyses of Figure 8c) in the MD simulations based on the distance between the carbon atom of the carboxyl group in PAA and the nitrogen of PDADMA. The cut-off distance for the pairing was determined as the distance of the first minima on the radial distribution function between the carbon atom of the carboxyl group and the nitrogen of PDADMA (0.6 nm).

Hydrogen bonds in Figure 6b and Figure S8 were calculated based on the donor-acceptor distance (0.35 nm) and angle (30°) between hydrogen-donor-acceptor pair.

References

1. Maryott, A. A.; Smith, E. R., *Table of dielectric constants of pure liquids*. US Government Printing Office: 1951; Vol. 514.
2. Martínez, L.; Andrade, R.; Birgin, E. G.; Martínez, J. M., PACKMOL: A package for building initial configurations for molecular dynamics simulations. *Journal of computational chemistry* **2009,** *30* (13), 2157-2164.
3. Berendsen, H. J. C.; Postma, J. P. M. v.; Van Gunsteren, W. F.; DiNola, A.; Haak, J. R., Molecular dynamics with coupling to an external bath. *The Journal of chemical physics* **1984,** *81* (8), 3684-3690.
4. Kastinen, T.; Batys, P.; Tolmachev, D.; Laasonen, K.; Sammalkorpi, M., Ion-Specific Effects on Ion and Polyelectrolyte Solvation. *ChemPhysChem* **2024**, e202400244.
5. Eneh, C. I.; Kastinen, T.; Oka, S.; Batys, P.; Sammalkorpi, M.; Lutkenhaus, J. L., Quantification of Water–Ion Pair Interactions in Polyelectrolyte Multilayers Using a Quartz Crystal Microbalance Method. *ACS Polymers Au* **2022,** *2* (4), 287-298.
6. Khavani, M.; Batys, P.; Lalwani, S. M.; Eneh, C. I.; Leino, A.; Lutkenhaus, J. L.; Sammalkorpi, M., Effect of ethanol and urea as solvent additives on PSS–PDADMA polyelectrolyte complexation. *Macromolecules* **2022,** *55* (8), 3140-3150.
7. Batys, P.; Kivistö, S.; Lalwani, S. M.; Lutkenhaus, J. L.; Sammalkorpi, M., Comparing water-mediated hydrogen-bonding in different polyelectrolyte complexes. *Soft Matter* **2019,** *15* (39), 7823-7831.
8. Suarez-Martinez, P. C.; Batys, P.; Sammalkorpi, M.; Lutkenhaus, J. L., Time–Temperature and Time–Water Superposition Principles Applied to Poly(allylamine)/Poly(acrylic acid) Complexes. *Macromolecules* **2019,** *52* (8), 3066-3074.
9. Eneh, C. I.; Nixon, K.; Lalwani, S. M.; Sammalkorpi, M.; Batys, P.; Lutkenhaus, J. L., Solid–Liquid–Solution Phases in Poly(diallyldimethylammonium)/Poly(acrylic acid) Polyelectrolyte Complexes at Varying Temperatures. *Macromolecules* **2024,** *57* (5), 2363-2375.
10. Batys, P.; Luukkonen, S.; Sammalkorpi, M., Ability of the Poisson–Boltzmann equation to capture molecular dynamics predicted ion distribution around polyelectrolytes. *Physical Chemistry Chemical Physics* **2017,** *19* (36), 24583-24593.
11. Bussi, G.; Donadio, D.; Parrinello, M., Canonical sampling through velocity rescaling. *The Journal of chemical physics* **2007,** *126* (1).
12. Bernetti, M.; Bussi, G., Pressure control using stochastic cell rescaling. *The Journal of Chemical Physics* **2020,** *153* (11).
